# Developmental vulnerability in children from culturally and linguistically diverse backgrounds in Western Australia: a population-based study

**DOI:** 10.1007/s12519-025-00936-0

**Published:** 2025-07-09

**Authors:** Kendalem Asmare Atalell, Gavin Pereira, Bereket Duko, Sylvester Dodzi Nyadanu, Vegard Skirbekk, Gizachew A. Tessema

**Affiliations:** 1 Curtin School of Population Health, Curtin University, Perth, WA Australia; 2 College of Medicine and Health Sciences, University of Gondar, Gondar, Ethiopia; 3 enAble Institute, Curtin University, Kent Street, Bentley, WA 6102 Australia; 4 Research Centre for Public Health, Equity and Human Flourishing (PHEHF), Torrens University Australia, Adelaide, SA Australia; 5 Healthy Environments and Lives (HEAL) National Research Network, Canberra, Australia; 6 Centre for Fertility and Health, Norwegian Institute of Population Health, Oslo, Norway; 7 School of Public Health, University of Adelaide, Adelaide, SA 5000 Australia

**Keywords:** Children development disparity, Culturally and linguistically diverse, Developmental vulnerability, Early childhood, Population-based cohort, Western Australia

## Abstract

**Background:**

Early childhood developmental adversities have long-term effects on educational and overall health outcomes. However, the developmental outcomes of children from culturally and linguistically diverse (CALD) backgrounds remain unclear. This study aimed to investigate the association between having a CALD backgrounds and developmental vulnerability in Western Australia.

**Methods:**

We conducted a retrospective population-based cohort study using data from the Australian Early Development Censuses, Midwives Notification System, and Hospital Morbidity Data Collections. Developmental vulnerability was defined as domain scores < 10th percentile in five Australian Early Development Censuses domains. Covariate-adjusted logistic regression, incorporating propensity score weighting, was applied, and the population attributable risk calculations results were informed.

**Results:**

Among 10,048 CALD children and 49,877 non-CALD children, 23.7% [95% confidence interval (CI) 22.9, 24.5%] of CALD children experienced developmental vulnerability in one or more Australian Early Development Censuses domain(s) (DV1) compared with 19.6% (95% CI 19.3, 20.0%) of non-CALD children. Adjusted analyses revealed that CALD children had 23% greater odds of DV1 [odds ratio (OR) 1.23; 95% CI 1.16, 1.31] and developmental vulnerability in two or more Australian Early Development Censuses domains (DV2) (OR 1.23; 95% CI 1.13, 1.33). CALD children had more than twice the odds of vulnerability in terms of communication skills and general knowledge (OR 2.16; 95% CI 1.99, 2.34) and 37% greater odds in language and cognitive skills (school-based) (OR 1.37; 95% CI 1.25, 1.51). The population attributable risk was 3.77% for DV1, 3.67% for DV2, 5.90% for language and cognitive skills (school-based), and 16.24% for communication skills and general knowledge.

**Conclusions:**

This study revealed a greater developmental vulnerability burden among CALD children than among their non-CALD peers, particularly in the domains of communication skills and general knowledge. Strengthening culturally responsive policies, enhancing early childhood support programs, and ensuring equitable access to educational resources for children from CALD backgrounds may help reduce developmental disparities and promote long-term educational outcomes.

**Graphical abstract:**

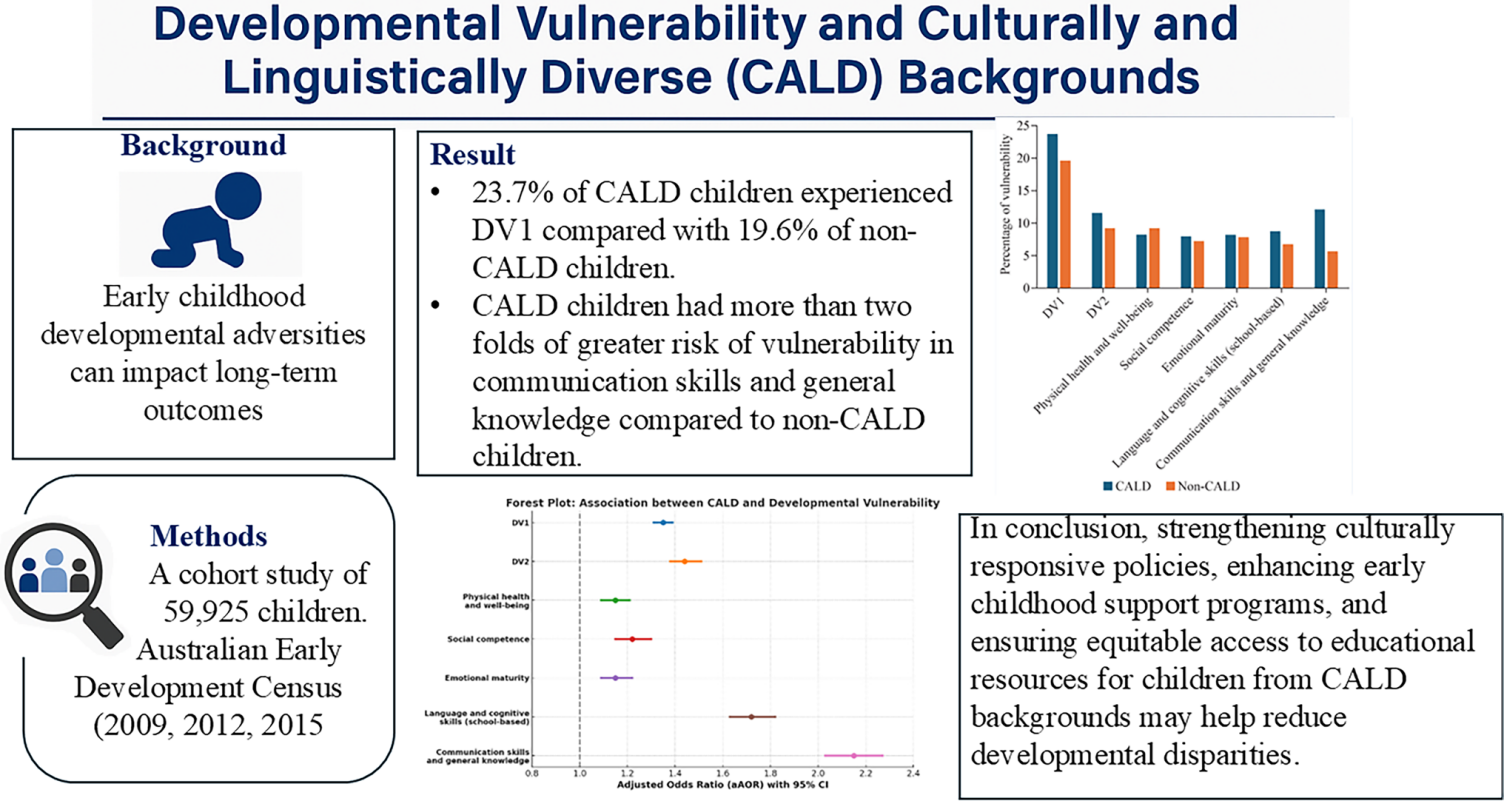

**Supplementary Information:**

The online version contains supplementary material available at 10.1007/s12519-025-00936-0.

## Introduction

Australia is a multicultural nation with a rich diversity of languages, cultural backgrounds and heritage. According to the 2021 Australia Population and Housing Census, over half of Australian residents were either born overseas or had parents who were born overseas, contributing to a broad range of linguistic and cultural backgrounds [1]. In Australia, the culturally and linguistically diverse (CALD) population, defined as individuals who do not belong to the mainstream English-speaking Anglo-Celtic group and are not aboriginal or Torres Strait Islander, is recognized as a priority population [2]. This diversity shapes various aspects of Australian society, including early childhood development, with children from CALD backgrounds facing significant challenges that may affect their development and well-being [3]. Language barriers may hinder communication and learning, whereas cultural differences may lead to feelings of isolation and difficulty adapting to new environments [4]. Economic disadvantages and housing instability further complicate their lives, limiting access to essential resources and contributing to stress. In addition, limited access to healthcare and the stress of resettlement can negatively impact physical and mental health, contributing to early childhood developmental vulnerability [5–9]. A study conducted in Spain also supports this, highlighting the impact of socioeconomic status on low birthweight between native and immigrant children, which further increases the risk of developmental adversities [10]. Similarly, a study in the USA recommended that educational, clinical, and family efforts to support the development of competence in both languages of dual-language children may prove rewarding, leading to long-term well-being, improved mental health, and cognitive and educational benefits [11].

Early childhood developmental vulnerability refers to the risk of a child not reaching their full developmental potential in critical areas necessary for success in school and later life [12, 13]. This includes delays or difficulties in physical health, emotional maturity, social competence, cognitive skills, language acquisition, and communication skills [13]. Children with developmental vulnerability may struggle with learning, social interactions, and overall well-being [12]. Understanding the disparities in early childhood developmental vulnerability among children is crucial, as it is a key indicator of future school success. Some of these disparities may be attributed to cultural and linguistic factors [5]. In addition, a study in Europe suggested that disparities in the growth and development of migrant and nonmigrant children are closely related to anthropological differences and socioeconomic status, highlighting the need for adequate healthcare for all groups [14].

In the Australian Early Development Census (AEDC) context, early childhood developmental vulnerability is assessed across five developmental domains: physical health and well-being; social competence; emotional maturity; language and cognitive skills (school-based); and communication skills and general knowledge. Previous studies have shown that children from CALD backgrounds experience poorer mental health outcomes at school entry than their Australian-born, English-speaking peers do [15]. A similar study conducted in the USA also indicated that language use at home, ethnic origin and maternal immigration status were associated with early childhood health and developmental outcomes [16]. However, studies on early childhood developmental vulnerability among those with CALD backgrounds are limited in Australia and globally. Given the existing evidence, we hypothesize that children from CALD backgrounds are more likely to experience developmental vulnerability at school entry than their non-CALD peers are. Thus, using state-wide, administratively linked datasets, we aimed to investigate the association between having a CALD background and developmental vulnerability at school entry (median age of five years) in Western Australia (WA).

## Methods

### Study design and setting

This retrospective cohort study compared developmental vulnerability among 10,048 CALD children and 49,877 non-CALD children at school entry, using data from Western Australia (WA).

### Data sources

This study utilized the administrative record linkage of the AEDC, the Midwives Notifications System (MNS) and the Hospital Morbidity Data Collection (HMDC). We used the first three consecutive AEDC collections (i.e., 2009, 2012 and 2015). The AEDC is a nationwide census of early childhood developmental vulnerability that assesses children in five key developmental domains: physical health and well-being, social competence, emotional maturity, language and cognitive skills (school-based), and communication skills and general knowledge. The census data were collected via the Australian version of the Early Development Instrument, which has been validated [17]. It is a comprehensive tool completed by teachers through a secured data entry system, which relies on their direct observations and knowledge of the children in their classes, combined with information from the enrollment forms. Teachers collected the data for children they had known for at least a month after receiving thorough training to ensure accuracy and consistency in the data collection process. The MNS dataset includes data related to childbirth, with records dating back to 1980, collected through notifications by midwives for births they attend in WA. The HMDC contains admitted patient data from public acute and psychiatric hospitals, private acute and psychiatric hospitals, and private day surgeries in WA. Established in 1970, it is one of the largest statutory data collections managed by the WA health system, increasing in size and complexity in line with population growth. The HMDC contains demographic and clinical information on services provided to admitted patients.

### Inclusion and exclusion criteria

**Fig. 1:**
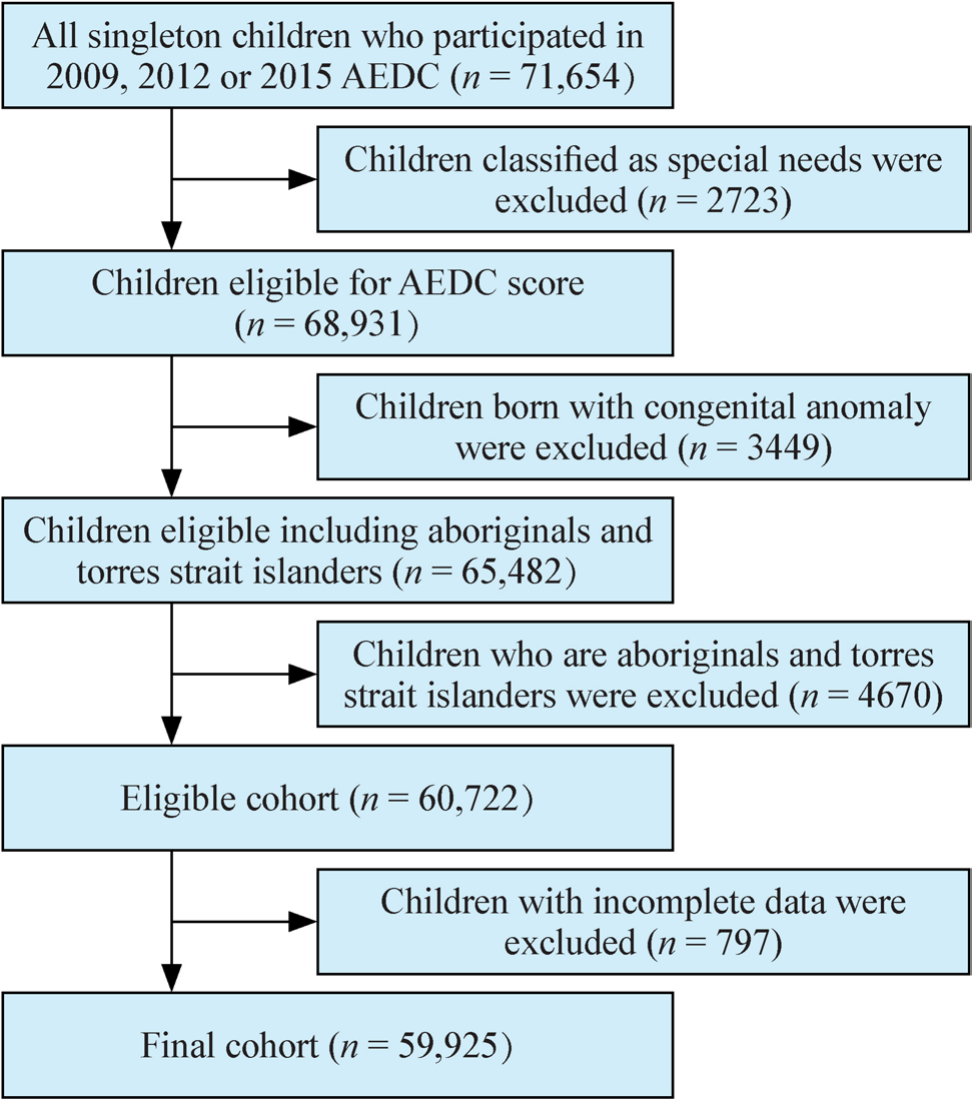
Flow diagram of the study. *AEDC* Australian Early Development Censuses, *DV1* vulnerability in one or more AEDC domain(s), *DV2* developmental vulnerability in two or more AEDC domains

This study included children who participated in the 2009, 2012 and 2015 AEDC collections. Children with congenital anomalies, special needs, incomplete AEDC values, and Aboriginals and Torres Strait islanders were excluded, resulting in a total of 59,925 children who remained in the analysis (Fig. 1).

### Outcomes

The primary outcomes included the two AEDC summary indicators of developmental vulnerability, as follows: DV1: developmentally vulnerable in one or more AEDC domain(s) and DV2: developmentally vulnerable in two or more AEDC domains. We also used the five AEDC developmental domains: physical health and well-being; social competence; emotional maturity; language and cognitive skills (school-based); and communication skills and general knowledge. Each domain is scored on a scale derived from the percentile ranking of the national population. Hence, children are classified into three categories: (1) developmentally on track if the child scores above the 25th percentile; (2) developmentally at risk if the child scores between the 10th and 25th percentiles; and (3) developmentally vulnerable if the child scores below the 10th percentile on the basis of reference cutoffs from the 2009 AEDC collection [18–20].

### Exposure

The exposure variable was children’s CALD background status, defined on the basis of criteria from previous literature [21]. This included speaking English as a second language, using a language other than English at home, being born outside of Australia or other major English-speaking countries, or being born to a non-Caucasian (i.e., child born to an Asian, African, Polynesian/Māori or other ethnic backgrounds) mother.

### Covariates

Sociodemographic risk factor data were obtained from the MNS and HMDC. These include child sex at birth (male, female), child age (< 5 years and 1 month, ≥ 5 years and 1 month to 5 years and 10 months, ≥ 5 years and 10 months), maternal age (< 20, 20–29, 30–39, ≥ 40 years), marital status (single, married, others), parity (nulliparous, primiparous, multiparous), index of relative sociodemographic disadvantage (< 20 percentile, 20–39, 40–59, 60–79, ≥ 80 percentile) and maternal tobacco smoking during pregnancy (yes, no). In addition, census year (2009, 2012 and 2015), birth season (spring, summer, autumn, and winter) and remoteness (very remote, remote, inner regional, and major cities) were used as covariates in our study.

### Statistical analysis

Given that missing data were not randomly distributed and accounted for less than 5% of the total dataset, we employed a complete case analysis to ensure robust and valid results. Descriptive statistics were used to compare demographic and maternal characteristics between children from CALD and non-CALD backgrounds. Logistic regression was used to assess the associations between CALD backgrounds and developmental vulnerability. Both unadjusted and adjusted odds ratios (ORs) with 95% confidence intervals (CIs) were calculated. Adjustments included census year, child sex at birth, child age, maternal age, marital status, parity, birth season, remoteness and socioeconomic index for areas. In addition, we added propensity score weighting to the main model to further account for residual confounders, improve robustness, and handle complex interactions. The propensity score was estimated via a logistic regression model using the covariates listed above. We calculated each observation’s stabilized inverse probability of treatment weights via the estimated propensity scores. Finally, the weights were applied in the main logistic regression model. The PAR with its 95% CI for each developmental outcome attributed to the CALD background was also calculated via the OR calculated from the above model.

The evidence shows that CALD varies across contexts [4]; therefore, we conducted sensitivity analyses considering the variables used to define CALD in our datasets, such as English as a second language, languages other than English at home, birth countries and maternal ethnic origins. To account for the effect of modifying role of sex at birth, we conducted a subgroup analysis for female and male children separately for DV1 and DV2 outcomes. We also conducted causal mediation analysis via the counterfactual framework, employing a parametric regression-based approach to assess the causal pathway of developmental vulnerability. This method builds upon conventional statistical mediation analysis, commonly known as the “Baron and Kenny procedure,” by accounting for exposure mediator interactions within the outcome regression model. It utilizes counterfactual definitions to estimate the natural direct effect (NDE) and natural indirect effect (NIE) through the mediator. Specifically, two models were developed: one modeling small for gestational age (SGA) (mediator) as a function of CALD (exposure) and covariates and another modeling DV1/DV2 (outcomes) as a function of the exposure, the mediator, and the covariates (Supplementary Figs. 1 & 2). All analyses were conducted via STATA 18.

## Results

Among the 59,925 children included in the analysis, 10,048 (16.8%) were classified as having a CALD background, and 51.1% of CALD and 50.4% of non-CALD children were male. The proportion of children with CALD backgrounds increased from 25.0% in 2009 to 41.4% in 2015. With respect to maternal age, 2.9% of CALD children and 3.7% of non-CALD children were born to mothers under 20 years of age. A greater proportion of CALD families than non-CALD families were in the lowest quintile (18.1% vs. 13.3%). In addition, low birth weight and preterm birth were more prevalent among CALD children than among non-CALD children. However, maternal smoking was less common in the CALD subgroup (7.2%) than in the non-CALD subgroup (14.6%) (Table 1).

**Table 1:** Characteristics of the study population by culturally and linguistically diverse background status (*n* = 59,925)

Variables^a^	CALD (*n* = 10,048)	Non-CALD (*n* = 49,877)
	*n* (%)	*n* (%)
Sex at birth		
Male	5137 (51.1)	25,156 (50.4)
Female	4911 (48.9)	24,721 (49.6)
Child age		
< 5 y and 1 mon	1854 (18.5)	8618 (17.2)
Between 5 y 1 mon and 5 y 10 mon	7407 (73.7)	36,935 (74.0)
> 5 y and 10 mon	787 (7.8)	4324 (8.7)
Year of AEDC collections		
2009	2511 (25.0)	16,092 (32.3)
2012	3372 (33.6)	18,042 (36.2)
2015	4165 (41.4)	15,743 (31.6)
Maternal age (y)		
< 20	294 (2.9)	1827 (3.7)
20–29	4292 (42.7)	20,923 (42.0)
30–39	5051 (50.3)	25,511 (51.2)
> 40	411 (4.1)	1616 (3.2)
Marital status		
Never married	565 (5.6)	3652 (7.3)
Married	9266 (92.2)	45,416 (91.1)
Other	217 (2.2)	809 (1.6)
Socioeconomic disadvantage for areas		
< 20th quantile (most disadvantaged)	1785 (18.1)	6461 (13.3)
20th–39th quantile	1979 (20.1)	8707 (17.9)
40th–59th quantile	2008 (20.4)	9518 (19.6)
60th–79th quantile	1990 (20.2)	11,779 (24.2)
> 80th quantile (least disadvantaged)	2085 (21.2)	12,179 (25.0)
Low birth weight		
Yes	485 (4.8)	1845 (3.7)
No	9563 (95.2)	48,032 (96.3)
Preterm birth		
Yes	613 (6.1)	2950 (5.9)
No	9424 (93.9)	46,901 (94.1)
Maternal smoking		
Yes	720 (7.2)	7276 (14.6)
No	9317 (92.8)	42,578 (85.4)

*AEDC* Australian Early Development Census, *CALD* culturally and linguistically diverse

^a^All categories showed statistically significant differences (*P* < 0.001) except for sex at birth (*P* = 0.208) and preterm birth (*P* = 0.463)

### Developmental vulnerability according to the culturally and linguistically diverse backgrounds

**Fig. 2:**
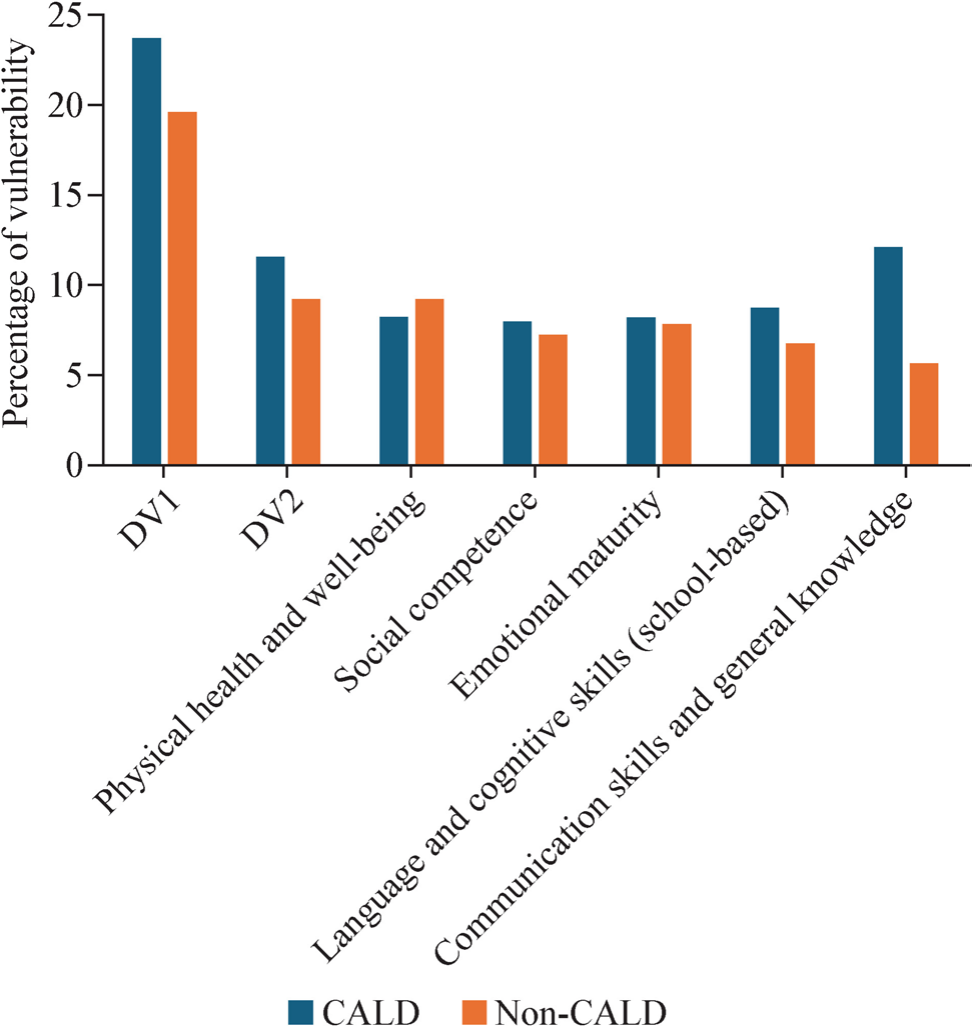
Developmental vulnerability according to the culturally and linguistically diverse background

The proportion of DV1 among CALD children was greater (23.7%) than that among non-CALD children (19.6%) (*P* < 0.001). Similarly, the proportion of DV2 was greater among CALD children (11.6%) than among non-CALD children (9.3%) (*P* < 0.001). With respect to developmental domains, vulnerability to communication skills and general knowledge was greater among CALD children (12.1%) than among non-CALD children (5.7%). In addition, CALD children exhibited greater vulnerability in language and cognitive skills (school-based) (8.8% vs. 6.8%), emotional maturity (8.3% vs. 7.9%), and social competence (8.0% vs. 7.3%). However, non-CALD children are more vulnerable in the domain of physical health and well-being, which highlights the notable contrast in the developmental challenges faced by the two groups (Fig. 2).

### Associations between culturally and linguistically diverse backgrounds and developmental vulnerability

The associations between developmental vulnerability and CALD status were assessed via logistic regression, adjusted for covariates and propensity score weighting. Children from CALD backgrounds had 23% greater odds of experiencing DV1 than non-CALD children did (OR 1.23; 95% CI 1.16, 1.31). Similarly, the odds of experiencing DV2 were also 23% greater among CALD children than among non-CALD children (OR 1.23; 95% CI 1.13, 1.33). Within the AEDC domains, children from CALD backgrounds had over two-fold higher odds of vulnerability in communication skills and general knowledge (OR 2.16; 95% CI 1.99, 2.34) and 37% higher odds in language and cognitive skills (school-based) (OR 1.37; 95% CI 1.25, 1.51). However, the odds of vulnerability in physical health and well-being were 17% lower for CALD children than for their non-CALD counterparts (OR 0.87; 95% CI 0.80, 0.96). In addition, vulnerability to social competence (OR 1.04; 95% CI 0.95, 1.14) and emotional maturity (OR 1.00; 95% CI 0.91, 1.10) were not significantly associated with CALD.

The PARs for DV1 and DV2 attributed to CALD were 382 (3.8%; 95% CI 2.64%, 4.95%) and 372 (3.7%; 95% CI 2.18%, 5.23%), respectively. A higher PAR attributed to CALD was observed in 1632 children (16.2%; 95% CI 14.23%, 18.33%) for communication skills and general knowledge, 593 children (5.9%; 95% CI 4.10%, 7.80%) for language and cognitive skills (school-based) and 71 (0.7%; 95% CI − 0.86%, 2.33%) for social competence. In contrast, the PAR for physical health and well-being was − 2.2%, indicating a negative effect of CALD on vulnerability in the physical health and well-being domain (Table 2).

**Table 2:** The association between culturally and linguistically diverse and developmental vulnerability at school entry

Outcome	OR	95% CI	^a^AOR	95% CI	PAR	95% CI
DV1	1.27	1.21, 1.34	1.23	1.16, 1.31	3.77	2.64, 4.95
DV2	1.28	1.20, 1.37	1.23	1.13, 1.33	3.67	2.18, 5.23
Physical health and well-being	0.89	0.83, 0.96	0.87	0.80, 0.96	− 2.19	− 3.53, − 0.76
Social competence	1.11	1.02, 1.20	1.04	0.95, 1.14	0.68	− 0.86, 2.33
Emotional maturity	1.05	0.97, 1.14	1.00	0.91, 1.10	0.00	− 1.49, 1.58
Language and cognitive skills (school-based)	1.33	1.23, 1.44	1.37	1.25, 1.51	5.90	4.10, 7.80
Communication skills and general knowledge	2.28	2.13, 2.45	2.16	1.99, 2.34	16.24	14.23, 18.33

*OR* Odds Ratio, *AOR* Adjusted odds Ratio, *CI* Confidence Interval, *PAR* Population Attributable Risk, *DV1* developmentally vulnerable on one or more AEDC domain(s), *DV2* developmentally vulnerable on two or more AEDC domains, *AEDC* Australian Early Development Census

^a^Adjusted for census year, child sex at birth, maternal age, marital status, parity, birth season, child age, remoteness and socioeconomic index for areas

Sensitivity analyses were conducted for DV1 and DV2 by incorporating small for gestational age, preterm birth, interpregnancy interval and maternal smoking into the main model individually and in combination. The model including maternal smoking increased the effect of CALD on DV1 (OR 1.28; 95% CI 1.21, 1.36) and DV2 (OR 1.30; 95% CI 1.19, 1.40) (Supplementary Table 1). In addition, we performed a sensitivity analysis to account for the varying definitions of CALD. Hence, children using English as a second language increase the odds of DV1 by 84% and DV2 by 74%. Children using languages other than English at home increased the odds of DV1 by 37% and DV2 by 30%. With respect to country of birth, children born from non-English-speaking countries had 41% greater odds of DV1 and 77% greater odds of DV2. With respect to maternal ethnic origins, children born to African, Polynesian/Maori and other ethnic origins increased the odds of DV1 by 35%, 31% and 30%, respectively. Similarly, children born to African, Polynesian/Maori and other ethnic origins increased the odds of DV2 by 44%, 45% and 27%, respectively (Supplementary Table 2). A comparison of the risk of being classified as DV1 or DV2 by sex at birth revealed an increased risk for females for DV1 (OR 1.34; 95% CI 1.22, 1.48) compared with males (OR 1.17; 95% CI 1.09, 1.27) (Supplementary Table 3).

In the causal mediation analysis, the natural direct effect estimates suggested that children with CALD backgrounds were more likely to be classified as DV1 (OR 1.23, 95% CI 1.17, 1.30) and DV2 (OR 1.24, 95% CI 1.15, 1.33) after adjusting for covariates. The effects of CALD on developmental vulnerability mediated by small for gestational age were 8.3% (6.9%, 9.3%) and 7.8% (6.2%, 8.8%) for DV1 and DV2, respectively (Table 3, Supplementary Figs. 1 and 2).

**Table 3:** Causal mediation analysis result on the influence of small for gestational age on the association between culturally and linguistically diverse and developmental vulnerability

Effects	DV1			DV2		
	^a^AOR	95% CI		^a^AOR	95% CI	
Controlled direct effects	1.23	1.17	1.30	1.24	1.15	1.33
NDE	1.23	1.17	1.30	1.24	1.15	1.33
NIE	1.02	1.01	1.02	1.02	1.01	1.02
Marginal total effects	1.25	1.19	1.32	1.26	1.17	1.35
Proportion of mediated by low birth weight = [NDE × (NIE − 1)]/(NDE × NIE − 1)	8.29%	6.87%	9.25%	7.83%	6.24%	8.79%

*AOR* Adjusted Odds Ratio, *CI* Confidence Interval, *DV1* developmentally vulnerable on one or more AEDC domain(s), *DV2* developmentally vulnerable on two or more AEDC domains, *AEDC* Australian Early Development Census, *NDE* natural direct effect, *NIE* natural indirect effect

^a^Adjusted for census year, child sex at birth, maternal age, marital status, parity, remoteness, birth season, age of the child and socioeconomic index for areas

## Discussion

This study aimed to assess developmental vulnerability among children from CALD backgrounds at school entry in Western Australia. Our findings showed that children from CALD backgrounds were more likely to experience developmental vulnerability than their non-CALD peers. These findings are consistent with those of previous studies, indicating persistent challenges faced by CALD children, particularly in the communication skills and general knowledge and language and cognitive skills (school-based) domains. These findings emphasize the need for targeted interventions and policies to support the developmental trajectories of CALD children and address disparities in early childhood development [15, 22]. The greatest disparities were found in vulnerability within the communication skills and general knowledge, as well as the language and cognitive skills (school-based) developmental domains. These disparities likely result from language barriers, cultural differences, and social isolation, which adversely affect early developmental outcomes and later educational trajectories [23]. Language barriers hinder academic performance and social integration, making it challenging for children to engage effectively in academic activities [24]. Cultural differences may contribute to mismatched educational expectations and reduced parental involvement, impacting children’s motivation and support at home [24]. In addition, social isolation may lead to emotional and behavioral issues, limiting peer relationships and informal learning opportunities and further exacerbating developmental vulnerability and academic challenges [25]. Furthermore, children from CALD backgrounds often experience lower socioeconomic status and limited access to health and early education and care services, as evidenced by previous studies [26, 27]. These barriers can delay the identification of developmental issues, hinder timely interventions and exacerbate disparities in developmental outcomes, particularly in language and cognitive skills (school-based) as well as communication skills and general knowledge [3]. Culturally responsive education, language support, and inclusive policies are essential to address these barriers. Targeted support through collaboration among health services, early childhood education providers, and nongovernmental organizations is vital for enhancing access to developmental surveillances and early interventions. These coordinated efforts can bridge developmental gaps and promote improved long-term developmental and educational outcomes for CALD children [2, 4].

On the other hand, this study revealed that children from CALD backgrounds presented lower developmental vulnerability in the physical health and well-being domains. This may be attributed to cultural practices emphasizing physical care, nutrition, and preventive health measures, which contribute to better health outcomes. In addition, strong familial support systems within CALD communities may play a key role in closely monitoring and addressing children’s physical health needs. A proactive approach to seeking medical care ensures timely interventions, supporting better physical development outcomes for CALD children [28]. Children from CALD backgrounds might benefit developmentally through bilingualism, which enhances cognitive flexibility, a cultural identity that supports emotional resilience, and access to broader social networks that foster social competence [29]. Furthermore, longitudinal studies are needed to explore broader developmental outcomes and whether these developmental gaps persist, widen, or narrow as children age through middle childhood and adolescence.

This study also revealed that the impact of CALD on developmental vulnerability was greater in females than in males. The greater impact of CALD on developmental vulnerability in females than in males may stem from cultural norms, differing socialization practices, and gendered expectations within CALD communities. Girls may face greater responsibilities at home, limited access to educational resources, and reduced social and cognitive development opportunities [30]. In addition, healthcare-seeking behaviors and teacher perceptions might favor boys, potentially delaying the identification of developmental challenges in girls [31]. Addressing these disparities requires culturally responsive strategies that promote gender equity in education, health access, and developmental support.

Despite recent initiatives, such as the Australian National Action Plan for the Health of Children and Young People 2020–2030, which identified CALD children as a priority population [32], developmental and educational challenges persist among CALD children. This indicates the need for targeted strategies and comprehensive support systems to address their unique needs and promote equitable outcomes [33]. The findings of this study have important implications for the development of policies and strategies aimed at improving early childhood developmental outcomes in CALD children [34]. Children from CALD backgrounds need access to culturally sensitive and inclusive early childhood education programs that address language barriers and ensure that they receive the necessary support [35]. Healthcare providers should also be attuned to the heightened risks faced by CALD populations, prioritizing early screening and interventions to address developmental challenges [34, 36]. The study also underlines the need for targeted interventions and culturally sensitive early childhood programs to address the developmental vulnerabilities faced by CALD children. While requiring initial financial commitment, such programs are cost effective in the long run because they reduce the need for remedial education and healthcare interventions. Effective strategies should address language barriers, promote cultural inclusivity, and ensure equitable resource allocation for vulnerable CALD populations. These may include bilingual educational resources, cultural competence training for educators, and the active engagement of CALD families in program design. Our study makes a unique contribution by specifically examining the developmental vulnerability of CALD children in Australia. Unlike previous research, it provides a comprehensive analysis of sociocultural influences on developmental outcomes while employing a novel methodological approach that integrates covariate-adjusted logistic regression and propensity score weighting.

The strength of this study lies in its pioneering use of linked data from multiple sources, including the AEDC, MNS and HMDC, to examine developmental vulnerability between children from CALD and non-CALD backgrounds. We also used covariate adjustment and propensity score matching to control for confounding factors. This study has several limitations. First, we relied on administrative linked data, which may lead to missing important covariates and affect the association between developmental vulnerability and CALD backgrounds. For example, maternal education and employment are important confounders. Nevertheless, we partly mitigate this limitation by adjusting for the socioeconomic index for statistical areas and remoteness, which is derived from income, education, employment, occupation, housing and access to resources at the area level. We also did not include the immigration status of the CALD population or the duration of residence in Australia, which might affect developmental vulnerability among CALD children. The other limitation of this study is that while CALD is a unique concept in Australia, there appears to be no uniform definition [21, 33]. Our study used proxy variables representing ethnic origin, language use and birth country. To account for this limitation, we have undertaken a sensitivity analysis by separately accounting for ethnic origin, language-related variables and birth country, which we used to define CALD.

In conclusion, this study identified disparities in developmental vulnerability at school entry between CALD and non-CALD children, with greater vulnerability in terms of communication skills and general knowledge, as well as language and cognitive skills (school-based) among CALD children. In comparison, non-CALD children exhibited greater vulnerability in terms of physical health and well-being. This finding emphasizes the need for targeted interventions for both groups. Collaboration among educators, policymakers, and CALD community stakeholders is essential to support all children with culturally responsive practices and additional resources for CALD families. Future research needs to investigate the mechanisms by which CALD and non-CALD children have disparities in developmental vulnerabilities, and longitudinal studies could better understand the long-term impact of early vulnerability on educational and social outcomes at later ages, such as middle childhood and adolescence. Validating AEDC tools for cultural sensitivity across diverse populations can help ensure their relevance for CALD children, as cultural biases in teacher-reported assessments may otherwise distort results.

## Supplementary Information

Below is the link to the electronic supplementary material.

Supplementary file1 (DOCX 123 KB)

## Data Availability

All data generated or analyzed during this study were included in this manuscript and are attached as supporting information.
