# Supplementary material for: Developmental vulnerability in children from culturally and linguistically diverse backgrounds in Western Australia: a population-based study
Source: World J Pediatr. 2025 Jul 9;21(7):744–54. doi: 10.1007/s12519-025-00936-0 (PMC12307503; doi:10.1007/s12519-025-00936-0)
Supplement: Supplementary file 1 — Supplementary file1 (DOCX 123 KB) [file 12519_2025_936_MOESM1_ESM.docx]

**Table S1.** Sensitivity analysis result of the association between CALD with DV1 and DV2 through adjusting for more confounding variables and propensity score weighting

| Outcome | A | | B | | C | | D | | F | | G | |
| --- | --- | --- | --- | --- | --- | --- | --- | --- | --- | --- | --- | --- |
|  | AOR | 95% CI | AOR | 95% CI | AOR | 95% CI | AOR | 95% CI | AOR | 95% CI | AOR | 95% CI |
| DV1 | 1.23 | 1.16, 1.31 | 1.22 | 1.14, 1.29 | 1.23 | 1.16, 1.31 | 1.22 | 1.15, 1.30 | 1.28 | 1.21, 1.36 | 1.25 | 1.17, 1.33 |
| DV2 | 1.23 | 1.13, 1.33 | 1.21 | 1.12, 1.32 | 1.23 | 1.13, 1.33 | 1.22 | 1.13, 1.33 | 1.30 | 1.19, 1.40 | 1.28 | 1.18, 1.39 |

*A: adjusted for census year, child sex at birth, maternal age, marital status, parity, birth season, child age, remoteness and socioeconomic index for areas*

*B: adjusted for covariates in model A and for small for gestational age*

*C: adjusted for covariates in model A and preterm birth*

*D: adjusted for covariates in model A and interpregnancy interval*

*F: adjusted for covariates in model A and maternal smoking*

*G: adjusted for covariates in model A and small for gestational age, Preterm birth, interpregnancy interval, and maternal smoking*

**Table S2.** Sensitivity analysis for the Association between developmental vulnerability (DV1 and DV2) and variables used to define CALD.

| **Outcome** | **Variables used to define CALD** | | **Unadjusted** | | **Adjusted** | |
| --- | --- | --- | --- | --- | --- | --- |
|  |  |  | **OR** | **95% CI** | **OR** | **95%CI** |
| DV1 | Using English as a second language | | 1.85 | 1.72, 1.98 | 1.84 | 1.68, 2.00 |
|  | Using language other than English at home | | 1.41 | 1.32, 1.50 | 1.37 | 1.27, 1.48 |
|  | Children categorised as CALD by using Ethnicity | | 1.30 | 1.22, 1.37 | 1.17 | 1.08, 1.47 |
|  | Ethnicity | Caucasian | 1.00 |  | 1.00 |  |
|  |  | Asian | 1.10 | 1.02, 1.18 | 1.06 | 0.96, 1.16 |
|  |  | African | 1.79 | 1.51, 2.13 | 1.35 | 1.10, 1.66 |
|  |  | Polynesian/Maori | 1.57 | 1.29, 1.91 | 1.31 | 1.03, 1.66 |
|  |  | Others | 1.52 | 1.39, 1.69 | 1.30 | 1.15, 1.47 |
|  | Country of birth | Children born from non-English speaking countries | 1.50 | 1.12, 2.00 | 1.41 | 1.00, 2.00 |
| DV2 | Using English as a second language | | 1.86 | 1.70, 2.03 | 1.74 | 1.56, 1.94 |
|  | Using language other than English at home | | 1.40 | 1.29, 1.52 | 1.30 | 1.18, 1.43 |
|  | Children categorised as CALD by using Ethnicity | | 1.31 | 1.21, 1.41 | 1.18 | 1.07, 1.30 |
|  | Ethnicity | Caucasian | 1.00 |  | 1.00 |  |
|  |  | Asian | 1.07 | 0.96, 1.19 | 1.05 | 0.92, 1.19 |
|  |  | African | 2.10 | 1.71, 2.58 | 1.44 | 1.13, 1.84 |
|  |  | Polynesian/Maori | 1.71 | 1.34, 2.19 | 1.45 | 1.08, 1.94 |
|  |  | Others | 1.50 | 1.32, 1.70 | 1.27 | 1.08, 1.49 |
|  | Country of birth | Children born from non-English speaking countries | 1.78 | 1.25, 2.53 | 1.77 | 1.14, 2.74 |

*Adjusted for census year, child sex at birth, maternal age, marital status, parity, remoteness, Birth season, age of the child and socioeconomic index for areas.*

**Table S3.** Subgroup analysis to assess the impact of CALD on developmental vulnerability (DV1 and DV2) for males and females.

| **Outcome** | **Sex** | **Unadjusted** | | **Adjusted** | |
| --- | --- | --- | --- | --- | --- |
|  |  | **OR** | **95% CI** | **OR** | **95%CI** |
| DV1 | Male | 1.22 | 1.14, 1.30 | 1.17 | 1.09, 1.27 |
|  | Female | 1.36 | 1.25, 1.48 | 1.34 | 1.22, 1.48 |
| DV2 | Male | 1.25 | 1.15, 1.36 | 1.20 | 1.09, 1.32 |
|  | Female | 1.35 | 1.19, 1.53 | 1.30 | 1.12, 1.50 |

*Adjusted for census year, child sex at birth, maternal age, marital status, parity, remoteness, Birth season, age of the child and socioeconomic index for areas*

**Table S4:** Causal mediation analysis result on the influence of small for gestational age on the association between CALD and developmental vulnerability (DV1 and DV2)

| **Effects** | **DV1** | | | **DV2** | | |
| --- | --- | --- | --- | --- | --- | --- |
|  | **Estimates** | **95% CI** | | **Estimates** | **95% CI** | |
| Controlled direct effects | 1.23 | 1.17 | 1.30 | 1.24 | 1.15 | 1.33 |
| Natural direct effects | 1.23 | 1.17 | 1.30 | 1.24 | 1.15 | 1.33 |
| Natural indirect effects | 1.02 | 1.01 | 1.02 | 1.02 | 1.01 | 1.02 |
| Marginal total effects | 1.25 | 1.19 | 1.32 | 1.26 | 1.17 | 1.35 |
| Proportion of mediated by low birth weight = [NDE × (NIE − 1)]/(NDE × NIE − 1) | 8.29% | 6.87% | 9.25% | 7.83% | 6.24% | 8.79% |

*Adjusted for census year, child sex at birth, maternal age, marital status, parity, remoteness, Birth season, age of the child and socioeconomic index for areas*


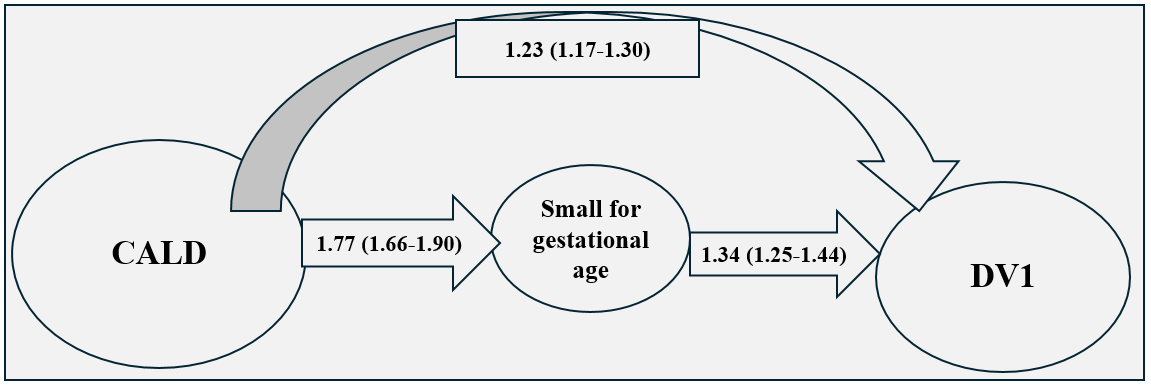


**Fig S1:** The influence of Small for gestational age on the association of CALD and DV1


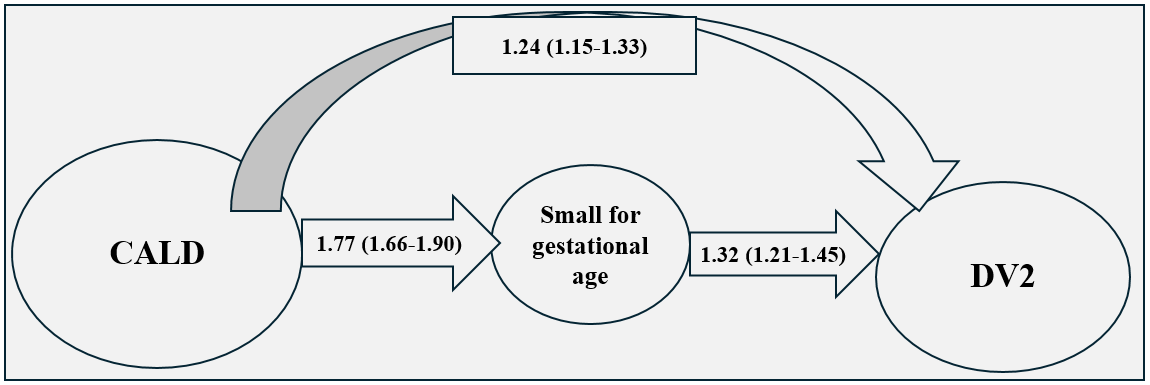


**Fig S2:** The influence of small for gestational age on the association of CALD and DV2
